# T cell receptor variable *β*20-1 harbors a nucleotide binding pocket in the CDR2*β* loop

**DOI:** 10.4236/oji.2013.33021

**Published:** 2013-09

**Authors:** Stephan Watkins, Werner J. Pichler

**Affiliations:** 1 Department of Rheumatology, Clinical Immunology and Allergology, Inselspital/University Hospital of Bern, Bern, Switzerland; 2 Department of Graduate Cell and Molecular Biology, University of Bern, Bern, Switzerland

**Keywords:** T Cell Receptor, T Cell Receptor Variable Domain, Adverse Drug Reactions, Autoimmune Diseases

## Abstract

Novel aspects of T cells containing TCRV*β*20-1 are numerous, ranging from pathogen specific reactivity to specific tissue homing, or possible T cell subsets. Recently, it was demonstrated that TCR itself could become reactive by binding to small molecules free of the pHLA interface. Our work here was to identify a natural ligand binding to an identified pocket on the CDR2*β* loop of these TCR. Using docking of suspected ligands, we were able to show Guanine and Adenine di- and tri-nucleotides readily bind to the identified site. Comparing these with small molecule sites found on other TCR types, we show this interaction is novel. With further molecular dynamic simulations, these sites are shown to be plausible by conducting simple computational based solubility tests as cross validation. Combined with simple proliferative responses, the identified nucleotides are also shown to have functional consequences by inducing T cell proliferation for CD4/V*β*20-1 + T cells, while failing to induce proliferation in other T cell isolates. Merging computational and simple cell assays, this work establishes a role of nucleotides in T cells found to contain this TCR sub-type.

## 1 Introduction

T cell receptors (TCR) themselves have been shown to contain novel binding sites on variable domains (TCRV) specific to subtype for a number of pathogenic proteins [1]. Recent work has also shown these can be extended to some small molecule interactions [2,3]. The established rolls for TCR subtypes based on TCRV have only recently been associated with adverse drug reactions (ADR) [4,5]. Many autoimmune diseases, however, have shown several associations with TCRV ranging from TCRV*β*20-1/ TCRV*α*17-1 in Sjögren’s syndrome, TCRV*β*5-1, 6 and 8 in Multiple sclerosis, or associated rheumatic diseases [6-10]. Additionally, specific T cell subsets have been identified with specific tissue homing, such as CD4/ TCRV*β*20-1 associated resident mucosal T cells [11,12]. Often the associations between diseases or ADR are difficult from literature alone, as despite international efforts different naming schemes still exist, such as with the TCRV*β*20-1, which may also be called TCR-MR, TCRV*β*2, or TCRV*β*2.1 as an example.

Here we focus on the TCR containing TCRV*β*20-1 as a novel TCR subtype able to illicit a response free from the peptide human leukocyte (pHLA) interface, classically represented as the main factor in disease models. Different hypotheses exist in both ADR and autoimmune diseases that overlap significantly [13,14]. In most of these, a myriad of factors exist making single causes problematic. In the entire receptor based mechanics, these often focus on the HLA subtype and the TCR-pHLA as the main factor. These over represented models rely often on other genetic factors, such as STAT4 promoter mutations in Sjögren’s syndrome, coupled with other genetic pre-dispositions such as HLA type associations [15-17]. This focus often identifies relevant HLA subtypes as the genetic associates, such as HLA-DRB4 in Rheumatoid arthritis, HLA-B*1502 in Carbamazepine drug allergy or both peptides derived from myelin basic protein peptide and HLA-DRB2 in multiple sclerosis [9,15,17-19]. These neglect factors from the TCR itself such as skewing associated with HLA type, which may be relevant for disease as well as initial targets to prevent disease. Additionally, these limit the search for other factors that may be involved, such as secondary small molecule co-factors.

Models of TCR only interactions which cause ADR have been described previously, termed non-covalent interaction with TCR [p-i TCR] models of drug induced allergy [20-22]. These postulate small molecules are able to illicit a response in the TCR by altering a site on these free of the pHLA interface. This model also implies there is no covalent modifications involved, nor interactions directly mediated from the HLA. In these models, the TCR triggering is still dependent on pHLA interactions for signaling in all cases. The small molecule itself only changes the TCR from a non-signaling to a signaling state through interactions on the TCR. Such disease models may simply be artifacts of pharmaceuticals and randomized amino acid sequences present on the TCRV themselves which vary between the 50 variable *β* or 45 *α*. However, these also may be indicative of a natural ligand-binding site that would show much higher frequency, especially for TCR containing such sites.

T cells positive for the TCRV*β*20-1 studied here have been isolated from Sjögren’s syndrome patients as a skewed responsive isolate from salivary glands in other studies [7,23]. This autoimmune disease involves many factors, initially an immunoglobulin (Ig) induced response, CD4 T cells then perpetuating a sustained inflammatory response. This leads to tissue damage, usually around the salivary glands, however including other sites as well. Tissue damage is the primary factor associated with these TCR, along with Th1 responses dictated by over expressed STAT4 as a secondary factor. As the primary Ig in all cases has been shown to target RhoGTPase, an additional factor has been the accumulation of GDP. This occurs throughout the body in Sjögren’s syndrome, however the disease manifests as sores in very specific sites [14,24-26].

Nucleotides themselves have proven to be problematic in respect to T cell responses, which show a heterogeneous response ranging from inhibitory expansion, to increased expansion. This difference in response has been identified as being related to P2x or P2y extracellular nucleotide receptors. These immune receptors were shown to be expressed in some CD4+ T cells, dendritic cells and macrophages [27-29]. Within the T cell pool, their expression is mixed, with some isolates showing no expression while others expressing one type. The expression on T cells has not been correlated with T cell sub-type other than CD4+, yielding literature with conflicting results as to T cell nucleotide proliferative responses.

Drawing on model autoimmune disease as a guide, nucleotides as extracellular signaling factors in inflammatory responses and sites found to be relevant on this TCR subtype in ADR models [2], we use computer models to identify possible natural ligands. Based on association of nucleotides, we use docking to test sites already identified as relevant on TCR to identify nucleotide binding sites. Additional computational work utilizing molecular dynamics is often used in rational drug design methodologies. Here we also utilize this method to test the validity of identified sites as significant in the same manner [30,31]. We aim to coordinate this computational approach to simple functional tests in T cell isolates, and work to develop a model relevant for both the autoimmune disease model and ADR as well. It is our aim to show overlap with these models through a minimal approach employing both techniques, which should be applicable to the immunological community as a whole.

## 2 Materials and Methods

### 2.1 TCRV*β*20-1 CDR2 Loop Sequence Alignments

The functional portion of TCRV*β*20-1, the entire CDR2 loop from residue 52 - 76, was blasted against the model organisms *X. leavis, D. rerio, M. musculus, B. tarus, H. sapiens, R. norvegicus*, using protein blast (http://blast.ncbi.nlm.nih.gov). Retrieved sequences were then aligned using clustalW (http://www.clustal.org). Models of the TCRV*α*7-1 and 20-1 were generated using Swiss model (http://swissmodel.expasy.org) [32] and the entire TCRVα with 4 alanine residues placed in the hyper variable regions between residue 95 and 99, and using PDB 3MV7 (http://www.pdb.org). These were further energy minimized in gromacs using force field 53a6, and SPC water at 300K for 2 ns [33,34].

### 2.2 Control T Cell Clone Isolation

The clone TCRV*β*5-1/Vα9-2, TCRV*β*20-1/Vα17-1 and TCRV*β*9/V α12-2 were isolated as described previously [22,35], by limited dilution. Briefly, whole blood mononuclear isolates from a patient with macula-papular eruptions to Sulfamethoxazole were first expanded with PHA, and subsequently isolated by limiting dilution. These were further isolated to single responsive T cell clones by cycles of expansion using only Sulfamethoxazole. Single clones were then sequenced for TCR type.

### 2.3 CD4/TCRV*β*20-1 Pull Down

Unlabeled Microbeads, CD4 labeled Microbeads and magnet were all purchased from MACS®Miltenyl Biotec. Unlabeled mouse anti human V*β*20-1 antibody was purchased from RayBiotech.Inc. Whole blood PBMC were isolated using a ficol gradient by centrifugation at 2000 RPM for 20 minutes, washing 2x in PBS and re-suspension in RPMI with 10% FCS. Immediately following, 100 Million cells added to CD4 attached beads according to MACS protocol, and separated using a bench-top magnet. These were then further enriched with V*β*20-1 (V*β*2 RayBiotech) and plated at 1000 TC to 10,000 irradiated allogeneic PBMC. These were expanded for 2 weeks, as described for cell expansion, again enriched for CD4 and V*β*20-1, and further expanded for 2 weeks before use. Cells were 99% CD4, V*β*20-1 by 4 weeks, as determined by FACS analysis.

### 2.4 Initial Structural Model Generation

Models of control or TCRV*β*20-1 containing TCR were generated against PDB 2NTS (*β*5-1), 3MV8 (V*β*9) or 2IJO (V*β*20-1) using Swiss model (http://swissmodel.expasy.org). Each of these has 96% homology to the respective variable domain. Additionally the CDR3 *α* or *β* respectively were α ALSNQAGT, *β* LLQGNTEA, *α* AVNFHSGN, *β* VDADTQYF and *α* ATDGNQF, *β* GQGENVY for control TCR models. These models were then energy minimized at 300 K in SPC water, force field 53a6 using gromacs for 1 ns. Resulting models were then utilized with all solvent removed. Small molecules, Sulfamethoxazole, GTP, GDP, cGMP, ATP, ADP, cAMP were generated in ACDlab-sChemsketch (Advanced Chemistry Development, Toronto, Ontario, Canada), converted to smiles format, and then PDB format generated using pymol (Schrodinger, LLC (2010) The PyMOL Molecular Graphics System, Version 1.3r1). Bond and angles were checked and corrected further in vacuo using gromacs, to generate final small molecule structures.

### 2.5 Docking of Nucleotide Analogues

Docking of Sulfamethoxazole, or derivatives of this compound have been reported previously to these same TCR or control TCR [2]. Nucleotide docking was performed using autodock vina [36,37], allowing residues in Sulfamethoxazole determined sites complete movement. This approach was to conduct 6 runs, each constituting 10 different dockings for the entire TCR. Additional restricted docking using 6 runs, with 10 different dockings was performed for sites already determined to bind Sulfamethoxazole. Nucleotide docking scores were then characterized as positive with a cutoff of –6.5 kcal/mol affinity as calculated by autodock vina.

### 2.6 Molecular Dynamic Simulations

Models for TCRV*β*20-1/V*α*17-1 used in docking were further energy minimized in gromacs using force field 53a6, embedded in SPC solvent, 0.14 M NaCl, 0.08 M KCl, 0.06 M MgCl and 0.04 M CaCl at 300 K, 1 ATM for 2 ns, also using initial docked conformations for SDM, SMX and GDP, and restraints. Simulations were then allowed to run for 10 ns simulation time, with V-rescale thermostat at 0.1 ps, and Paranillo-Rahman pressure coupling at 1 ps. The TCR was restrained using default Gromacs positional restraints, while each small molecule was unrestrained. Distances between protein residue TYR*β*58 and small molecule centers of mass (COM) were calculated over the entire 10 ns using Gromacs. Means were calculated in qtiplot (Copyright © 2004-2011 Ion Vasilief and Stephen Besch) as a moving window average.

### 2.7 T Cell Clone Proliferation Assays

Proliferation assays were conducted as described elsewhere [22]. In short, 25,000 TCC and 30,000 irradiated PBMCs or EBV-BCLC were incubated together per well with 200 μmol/ml of the indicated nucleotide or other compounds, medium alone (CM) or PHA (1 ug/ml). ^**3**^H-thymidine was added after 48 for exactly 12 hrs and proliferation measured via scintillation counting (Top Count, Perkin Elmer). For Sulfanilamide profiles shown, compounds were used at 250 μg/ml final concentrations [2]. Averages of at least three and up to six runs for each experimental condition were collected and all means along with statistical differences calculated. EBV-BCLC, positive for HLA-DRB10 were used for sulfanilamide cross reactivity profiles, while autologous PBMC were used in nucleotide assays.

### 2.8 Statistics

All Statistics were performed using qtiplot (Copyright © 2004-2011 Ion Vasilief and Stephen Besch). Proliferation data was fed into the software from spread sheets as three independent wells across three different experiments, and standard error, means and standard deviations plotted for individual points in all graphs. One way ANOVA was performed on plotted data, and no significance-scored as P-values > 0.05, significance, <0.05, *, or <0.01, **.

## 3 Results

### 3.1 Sequence Alignments

**Figure 1:**
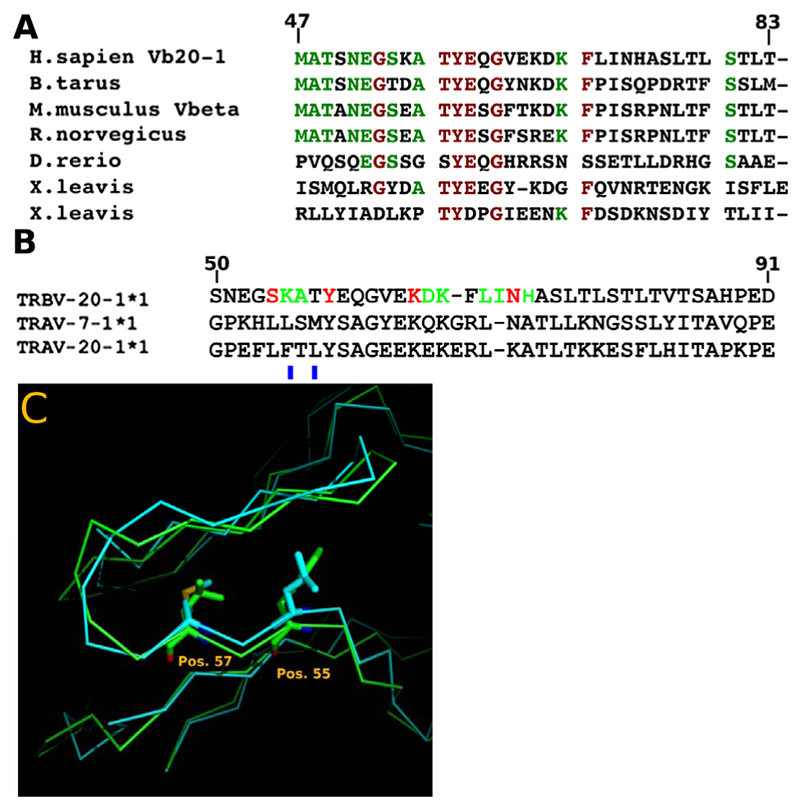
Sequence alignments. (A) Comparison of the CDR2 loop of human TCRV*β*20-1 with representative organisms. Green letters represent >60% conservation and Red, >85% across species. The loop is highly conserved across mammalian species, with high similarity even across distant species such as X. leavis. X. leavis retains two close matches, while other organisms contain one. (B) Comparison of human related TCRV with significant similarity. Only two TCRV*α*, 7-1 and 20-1, show any homology. Red letters, amino acid hydrogen bond with ligands, Green, backbone hydrogen bonds with ligand. Both show mutations rendering the loop incapable of binding nucleotides, indicated with blue bar underneath. (C) Alignments of the two human TCRV*α* showing overlapped K- > L/F at position 55, and T- > M/L at position 57. Mutated residues occupy most of the loop pocket.

Studies involving TCR evolution have been conducted several times by a number of groups [38,39]. Comparison of conserved amino acid sequences can be used to illustrate functional from non-functional residues or domains in proteins [38,40]. By comparing cross species sequences only incorporating the TCRV*β*20-1 CDR2 loop, a high level of conservation is observed (Figure 1(A)). This is in contrast to other data using TCR as a whole, which have illustrated conserved structures without conservation of other variable domain residues making up the various loops. This is further highlighted by comparing the same CDR2 loops within only H.sapiens, with similar TCR believed to represent recent evolutionary gene duplications (Figure 1(B), (C)). In the two closely related TCR, both are found in the TCRV*α* pool. For both of these, mutations highlighted indicate a rapid loss of the site by residues that fill the pocket formed in the CDR2*β* loop. Together, these alignments suggest a long evolutionary conservation of the loop as it is present across distantly related species with all amino acids found to be important for ligand binding in prior studies [1,2]. This is even truer for mammalian species, where the loop is almost identical. The fact that any closely related TCRV in humans render the site inaccessible also highlights a possible novel roll for this CDR2 loop.

### 3.2 Initial Structural Models

**Figure 2:**
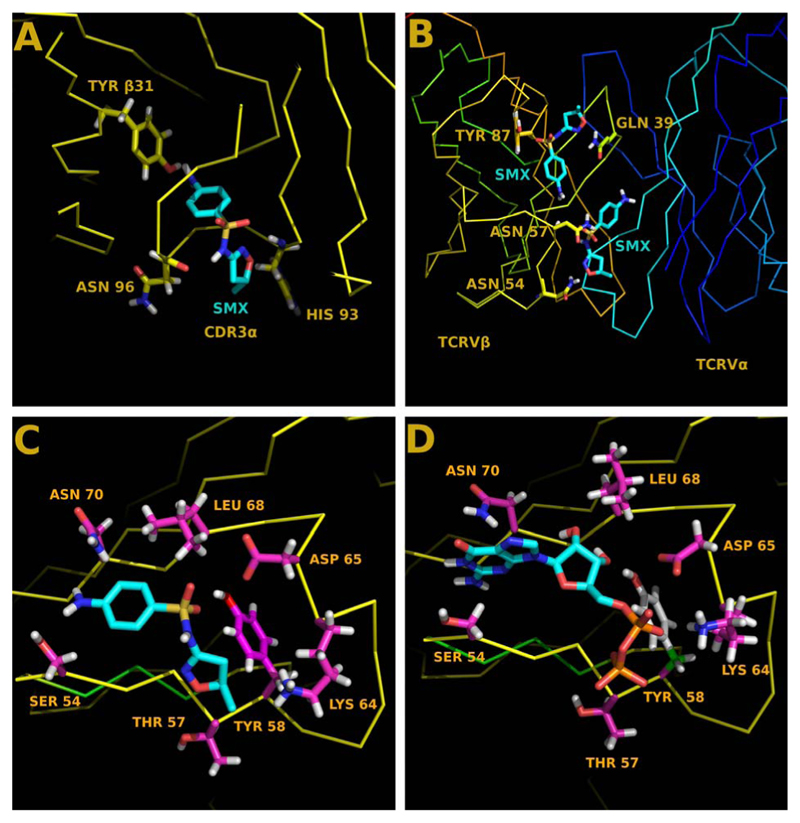
Docked ligands on TCR models. (A) Control TCRV*β*9/V*α*12-2 with bound SMX in CDR3*α* loop (B) Control TCRV*β*5-1/V*α*9-2 with bound SMX to two sites on the TCRV*β* domain. Yellow, V*β* Blue Vα. (C) TCRV*β*20-1/V*α*17-1 with bound SMX in the CDR2*β* loop. (D) Same as (C), with GDP bound in same pocket. Residues shown in (C), (D) make up part of the ligand binding pocket. In (A), (B) residues shown are for orientation of the TCR to the viewer. (A) and (C) both represent data shown in ref [2].

Initial models were based on prior docking experiments using SMX or related sulfanilamide compounds. These are shown here in Figures 2(A)-(C), and are primarily used as controls to illustrate differences found between TCR. Clear differences are observed in docking patterns which either show expected CDR3 loop recognition (Figure 2(A)) of compounds shown to cause ADR, or represent novel sites which may have further relevance (Figures 2(B) and (C)). In all cases, further work is necessary to delineate real versus non-significant docked sites. This is highlighted with the TCRV*β*20-1, where similar non-activating compounds have only very subtle differences in docking conformations from activating compounds.

### 3.3 Docking of Nucleotides

Based on association with V*β*20-1/V*α*17-1/CD4 + T cells with Sjögren’s syndrome tissue damage, we chose to coordinate nucleotide analogues with TCR bearing SMX docked sites. These were limited to GTP, GDP, cGMP, ATP, ADP, and cAMP as a representative pool. Control TCR not containing TCRV*β*20-1, but harboring strong affinity for SMX failed to dock any nucleotide analogues. Interestingly the CDR2*β* loop of the TCRV*β*20-1 readily docked GTP, GDP, ATP and ADP, but not either cyclic compound based on a cutoff of –6.5 kcal/mol. For GTP and GDP the affinity was –8.5 kcal/mol, which was much higher than SMX at –6.5 kcal/mol, however the same residues were involved with binding all nucleotides (Figure 2(D)). In all cases, the TYR58 was the highest energy bond formed, at 1.8 Å between TYR58 OH and O3’ of the nucleotide sugar ring. With nucleotide docking, unlike SMX, there was an additional interaction with the backbone of HIS71, mostly from size differences in the small molecules. This entailed a hydrogen bond with the guanine or adenine terminal NH2 and the amide O of HIS71.

### 3.4 Sulfanilamide Proliferation Assays

**Figure 3:**
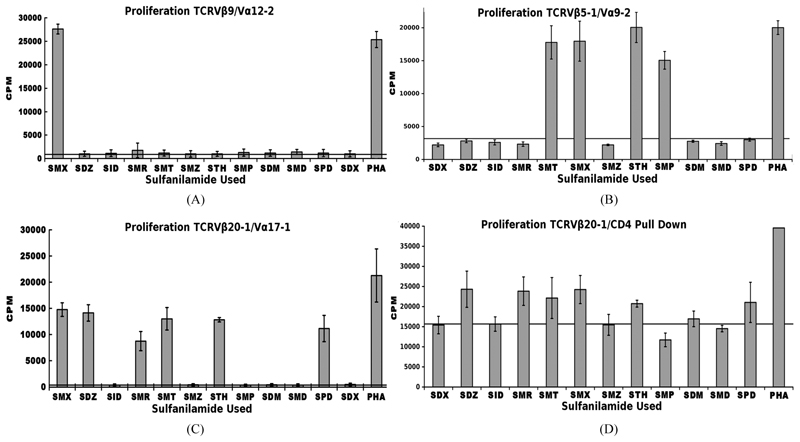
Sulfanilamide Proliferation Responses. (A) TCRV*β*9/V*α*12-2 sulfanilamide cross reactivity, (B) TCRV*β*5-1/V*α*9-2 cross reactivity, (C) TCRV*β*20-1/V*α*17-1 cross reactivity, and (D) TCRV*β*20-1/CD4+ pull down pool cross reactivity. Solid second x-axis line across graphs represents background cut off. In all EBV-BCLC were used as APC, which were non-autologous for the pull down pool. Shown are means of triplicates experiments with three wells each experiment, grey bars, with STDV as black error bars. (A), (C) were both shown as figure in prior work [2].

We previously conducted Sulfanilamide proliferation assays for 12 compounds, inclusive of SMX (Figure 3). This showed a particular patter for a control TCRV*β*20-1/V*α*17-1 + T cell clone (Figure 3(C)). To further correlate a generalized V*β*20-1 profile, and to add significance to our V*β*20-1/CD4 pull down cell pool, these were also subject to the same proliferation assay (Figure 3(D)). Not shown, we also conducted the same assay with only V*β*20-1 pull downs, heterogeneous for CD4/CD8, which contained a significant background. These showed only conclusive response from SMX above a CPM background of 15,000. For the double pull down, a clear pattern matching the original single T cell isolate was determined. This also shows a significant background due to heterogeneity in TCRVα, however demonstrate a uniform response between CD4/V*β*20-1 double positive phenotypes. Additionally, the experimental set up utilized Epstein bar virus immortalized B cells specifically containing HLA DRB10, to make sure the experiments were all conducted exact. These were non-autologous for the pull down CD4/TCRV*β*20-1 pool.

### 3.5 Molecular Dynamics of TCRV*β*20-1

**Figure 4:**
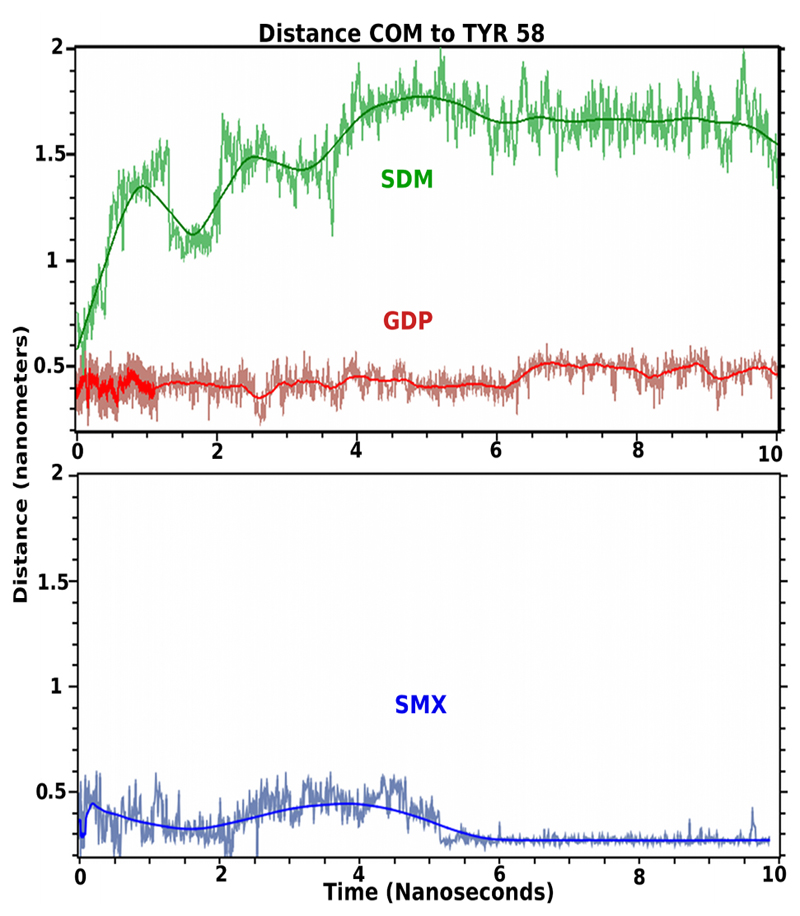
TCR bound ligand molecular dynamic simulation in solvent. Shown are 10 nanosecond simulations of indicated ligand starting from docked conformations in the CDR2 loop of TCRV*β*20-1. Colored line coordinates to mean distance from Center of mass (COM) of small molecule from TYR58 as central residue in the pocket. Light fluctuating lines are actual position at 5 picosecond intervals. Both SMX, blue and GDP, red, remain bound in the pocket, and SMX adopts a slightly tighter bound conformation within the pocket at 5.5 nanoseconds. SDM, green, leaves the pocket almost immediately.

Molecular dynamics is used as a type of solubility test further validating docked small molecules in rational drug design strategies [30,31]. These allow the effects of water and ion mixtures, along with temperature to be incorporated into the computational design, or behavior of sites of interest. These also allow the time of occupancy for a small molecule for a particular protein site to be determined, which is missing from docking analysis alone. Using a standard simulation set up, the initial docked conformations of GDP, SMX and Sulfadimeth-oxine (SDM), a sulfanilamide found to not stimulate T cells containing TCRV*β*20-1, were allowed to run for 10 ns (Figure 4). This later compound was used as a negative control, while SMX served as a positive control based on cross correlated docking and proliferation.

Both SMX and GDP remain in the CDR2*β* loop, while SDM moves from the pocket almost immediately, however rolls on the surface of the TCR or becomes solvated transiently. Visually, the SDM sulfanilamide-substituted group hinders the SO2 and terminal NH2 of the sulfanilamide core from properly orienting itself within the CDR2*β* loop, while also allowing greater interactions overall with solvent. This illustrates a very fine specificity for not only the CDR2 loop, but also any site that may show a difference for closely related compounds differing by only small angles or distances at the atomic level. For SMX and GDP, the main interaction is with the sulfanilamide core, or the nucleotide core, also inclusive of the SO2 or first PO2 group and the 3’ O of the sugar ring. Both SMX and GDP slightly reoriented positions, around 0.8 - 1 ns and 5.5 ns. For GDP this is inclusive of HIS 71 interactions with the terminal NH2 of the nucleotide. This secondary computational analysis indicates the docking software properly found docked versus nondocked compounds, even with closely related SDM and SMX. Additionally, a dynamic process is observed with hydrogen bonding within the pocket alternating between closely spaced residues and loop backbone atoms. Through the entire simulation, hydrogen bonding with TYR58 is maintained for both bound molecules.

### 3.6 Nucleotide Induced Proliferation

**Figure 5:**
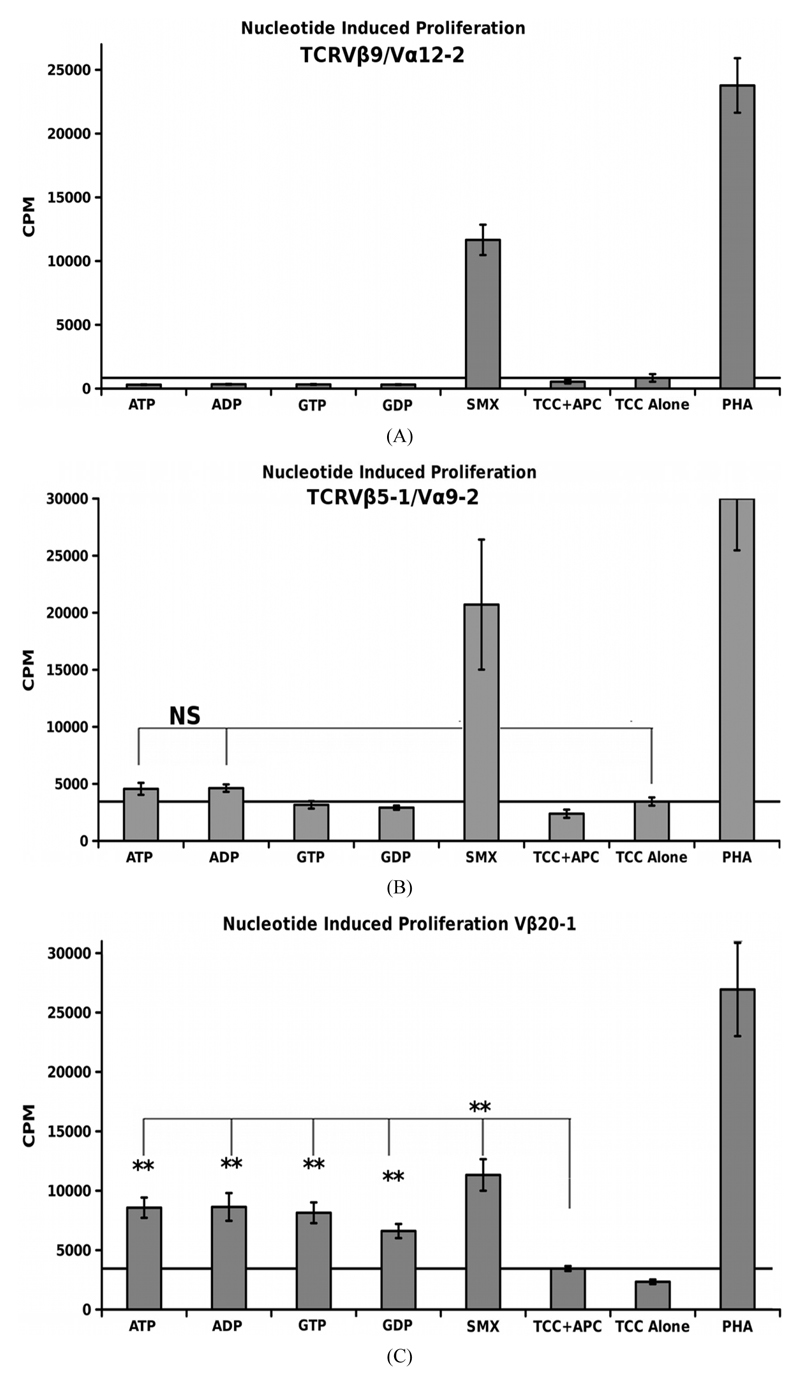
Nucleotide Induced Proliferation. (A) TCRV*β*9/V α12-2 nucleotide proliferation (B) TCRV*β*5-1/Vα9-2 nucleotide proliferation and (C) TCRV*β*20-1/CD4 + pull down nucleotide proliferation. In all, SMX is used also as a positive control, along with PHA. In (B), a slight response above background was noted, but not significant. Solid secondary x-axis line in all across graph indicates background. Non-continuous line in (B), (C) shows background column used for 1 way ANOVA significance tests for each individual nucleotide, or SMX in (C). Each is triplicate experiments with three wells each experiment, means, grey bars, STDV, black error bars. Autologous APC were used for each.

A simple nucleotide proliferation assay was conducted for TCRV*β*5-1/V*α*9-2, TCRV*β*9/V*α*12-2 monocultures and the CDR4/TCRV*β*20-1 pull down pool (Figure 5). This later remains heterogeneous for the TCRVα used by cells. Proliferation profiles show nucleotides ATP, ADP, GTP, GDP and SMX as a positive control to test for stimulation capacity. Clones containing TCRV*β*5-1/*Vα9-*2 or TCRV*β*9/V*α*12-2, had shown a proliferative response to SMX, and sulfanilamide derivatives SMT, STH, and SMP or only SMX respectively but also have differences in background obtained. Significant proliferation was shown only in the CDR4/TCRV*β*20-1 pull down pool for any of the nucleotides tested, p-values < 0.01, however the binding affinities from docking did not correlate to strength of proliferative response. The proliferative response itself is well above background, but less than SMX. This indicates adenine and guanine-di or -tri nucleotides themselves can act as ligands, inducing proliferation in T cells containing V*β*20-1. For the control clonotype TCRV*β*5-1/V*α*9-2, a very weak response is observed for ATP and ADP not mirrored with the TCRV*β*9/Vα12-2. For either nucleotide respectively, the p-values were 0.14 and 0.058, but highlight differences observed in the control pool well. These responses together reflect the confounding data represented in literature regarding CD4+ T cell nucleotide responses, with the CD4/V*β*20-1 + pool well above the weak responses reported for cells responding to ATP alone [27-29]. Overall, this indicates the docking software is able to determine correct interacting sites on TCR.

## 4 Discussion

Work presented here builds on an initial study designed to characterize non-classical TCR mediated drug allergies to sulfanilamides, in particular SMX. For most of these ADR, significant overlap exists with autoimmune disorders, with ADR even being referred to as the imitator of disease [41]. In either, there are mitigating factors involved, often due to the number of different components involved. These range from multiple cell types to differences in similar cells behavioral responses, or even varied chemokines produced by the same cells under different stimulus [14,18,42,43]. Our aim was to utilize determined sites relevant for ADR that show hyper proliferative responses in T cells and correlate these with a disease model associated with our developed models. This entailed both a V*β*20-1 association, along with autoimmune disease found to bear hyper proliferative T cells. From literary review, we found Sjögren’s syndrome overlapped somewhat with prior work. Underlying this, we sought to determine other mitigating aspects of the disease model that could be highlighted by the ADR work already conducted.

Based on this, we conducted nucleotide docking across TCR containing variable domains *β*20-1 and *α*17-1, along with control TCR also found to bind to SMX with high affinity. Our initial computer work showed TCRV*β*20-1 specificity for guanine and adenine di- and tri-nucleotides, while failing to bind to cyclic mono phosphate analogues. While neither control TCR bound any nucleotides tested, other TCR in the entire TCR repertoire may harbor sites not tested. T cells containing the two control TCR, and T cell pools typed only for TCRV*β*20-1/CD4 also correlated with the docking. This also demonstrated GTP, GDP, ATP and ADP as ligands capable of causing hyper proliferation with T cells containing variable domain *β*20-1.

Correlated with Sjögren’s syndrome this particular aspect of T cells containing this *β* subtype, possibly furthered by α subtype as well, has significance [7,44]. One aspect of ADR is the wide spectrum of responses observed, which can vary depending on the compound involved. The compound here, SMX, has shown a range of responses from Steven’s-Johnson syndrome to simple papular eruptions, with no direct correlation [45,46]. Other compounds such as Carbamazepine show either skewed TCR subtypes or HLA type associations [4,17,42]. Underlying aspects, such as a particular binding site on T cell subsets for a natural ligand, which subsequently recognizes a foreign compound as well could explain some differences.

In Sjögren’s syndrome, both STAT4 up regulation due to promoter mutations and often similar up regulation of basement membrane proteins such as Lamaninα2 are a pre-defining factor [24,26]. As a possible mitigating factor, guanine nucleotides would be abundant, as most patients also show Ig produced against RhoGTPases, molecules that would target most cells in the body [14]. Adenine nucleotides have been shown to play modulator rolls in CD4+ T cell responses, and the elevated abundance of either nucleotide in damage tissue would make them likely candidates for multiple targets effecting responses [27]. Mucosal specific T cell subsets have been characterized in a small pool of studies, showing a TCR homogenous for variable *β*20-1 that also overlaps with resident skin CD4+ T cells, or Sjögren’s isolated T cells, also marking them as possible similar responsive subsets [11,12]. This may explain the focalized aspect of both autoimmune disease involving any of these subsets of cells, and often SMX induced ADR involving papular or focalized mucosal eruptions.

While further work would be required to define these aspects in more detail, the findings here illustrate the use of combined computational and direct cellular research techniques to other disease-based models and ADR. As nucleotides were directly shown to be involved with these TCR as a previously undefined mitigating factor, research into ADR pathologies can be shown to open up new possibilities for not only disease models, but also possibly more precise disease treatment strategies. These strategies should foster such techniques when practical, for instance, targeting specific T cell subsets based on only TCRV*β* or α repertoire. Already techniques are employed with generalized phenotype approaches, such as modulation of Th17 responses or vaccination in multiple sclerosis against the CDR2 loop of TCRV*β*5-1 [47,48]. Even more precise targeting in autoimmune disease would increase safety aspects associated with immune manipulation in general by decreasing functional loss of a portion of immune protection to a minimum achievable point.

## 5 Conclusion

T cells double positive for TCRV*β*20-1/CD4 proliferate in the presence of guanine and adenine nucleotides when antigen-presenting cells alone are present. The identified binding site is free of the pHLA interface, and correlates with an SMX determined site previously found to cause hyper proliferation in ADR models. This site itself is an evolutionary conserved CDR2 loop of this variable domain, a site free of TCR hyper variable re-arrangement found in the normal TCR restriction process. Because of this conserved aspect, T cells positive for TCRV*β*20-1 have a clear roll in both allergic responses to compounds also found to bind at this site, and also as players in diseases associated with T cell subsets always positive for this variable domain. Additionally, the TCR here adds a further role for free extracellular nucleotides to the entire inflammatory response through this mechanism of proliferative response, which differs from other known nucleotide based immune cell responses.

## Abbreviations

TCR: T cell receptor
TCRV: TCR variable domain
ADR: Adverse drug 553 reactions
SMX: Sulfamethoxazole
SDM: Sulfadimethoxine
V*α*/*β*: TCRV alpha/beta
Ig: Immunoglobulin
